# Association of Prenatal Exposure to Valproate and Other Antiepileptic Drugs With Risk for Attention-Deficit/Hyperactivity Disorder in Offspring

**DOI:** 10.1001/jamanetworkopen.2018.6606

**Published:** 2019-01-04

**Authors:** Jakob Christensen, Lars H. Pedersen, Yuelian Sun, Julie Werenberg Dreier, Isabell Brikell, Søren Dalsgaard

**Affiliations:** 1 Department of Neurology, Aarhus University Hospital, Aarhus, Denmark; 2 Department of Clinical Epidemiology, Aarhus University Hospital, Aarhus, Denmark; 3 The National Centre for Register-Based Research, Department of Economics and Business Economics, Business and Social Science, Aarhus University, Aarhus, Denmark; 4 The Lundbeck Foundation Initiative for Integrative Psychiatric Research, iPSYCH, Aarhus, Denmark; 5 The Centre for Integrated Register-Based Research (CIRRAU), Aarhus University, Aarhus, Denmark

## Abstract

**Importance:**

Valproate is an antiepileptic drug (AED) used in the treatment of epilepsy and many other neurological and psychiatric disorders. Its use in pregnancy is associated with increased risks of congenital malformations and adverse neurodevelopment in the offspring and may be associated with an increased risk of attention-deficit/hyperactivity disorder (ADHD).

**Objective:**

To determine whether prenatal exposure to valproate and other AEDs is associated with an increased risk of ADHD in the offspring.

**Design, Setting, and Participants:**

This was a population-based cohort study of all live-born singleton children in Denmark from January 1, 1997, to December 31, 2011 (N = 913 302). Information on prenatal exposure to AEDs, including valproate, was obtained from the Danish National Prescription Registry and all children with ADHD were identified (children with diagnosed ADHD in the Danish Psychiatric Central Research Register or children who redeemed a prescription for ADHD medication). The cohort was followed up from birth until the day of the ADHD diagnosis, death, emigration, or December 31, 2015, whichever came first. Data were analyzed in September 2018.

**Exposures:**

Maternal use of valproate and other AEDs in pregnancy.

**Main Outcomes and Measures:**

Cox regression estimates of the hazard ratio of ADHD. Estimates were adjusted for potential confounders.

**Results:**

The cohort included 913 302 children (mean age at end of study, 10.1 years; median age, 9.4 years; interquartile range, 7.2-12.8 years; 468 708 [51.3%] male). A total of 580 were identified as having been exposed to valproate during pregnancy; of them, 49 (8.4%) had ADHD. Among the 912 722 children who were unexposed to valproate, 29 396 (3.2%) had ADHD. Children with prenatal valproate exposure had a 48% increased risk of ADHD (adjusted hazard ratio, 1.48; 95% CI, 1.09-2.00) compared with children with no valproate exposure. The absolute 15-year risk of ADHD was 4.6% (95% CI, 4.5%-4.6%) in children unexposed to valproate and 11.0% (95% CI, 8.2%-14.2%) in children who were exposed to valproate in pregnancy. No associations were found between other AEDs and ADHD.

**Conclusions and Relevance:**

Maternal use of valproate, but not other AEDs, during pregnancy was associated with an increased risk of ADHD in the offspring. These findings have important implications for the counseling of women of childbearing potential using valproate.

## Key Points

**Question:**

What is the risk of attention-deficit/hyperactivity disorder in children prenatally exposed to valproate and other antiepileptic drugs?

**Findings:**

In a population-based cohort study of 913 302 children in Denmark, prenatal exposure to valproate was significantly associated with a 48% increased risk of attention-deficit/hyperactivity disorder compared with children with no valproate exposure. No association was identified for other antiepileptic drugs.

**Meaning:**

The findings of this study corroborate that counseling is appropriate for the use of valproate in pregnancy and in women of childbearing potential.

## Teaser

This population-based cohort study of children in Denmark evaluates whether maternal use of valproate or other antiepileptic drugs during pregnancy is associated with the risk of attention-deficit/hyperactivity disorder (ADHD) in offspring.

## Introduction

Antiepileptic drug (AED) exposure during pregnancy is associated with an increased risk for congenital malformations^1,2^ and delayed cognitive development in the offspring.^3^ However, to our knowledge, only 5 small studies and a meta-analysis of these studies including a total of 816 persons with attention-deficit/hyperactivity disorder (ADHD) have examined a possible association between prenatal exposure to valproate and ADHD in the offspring.^4,5,6,7,8^

Although the heritability of ADHD has been estimated to be 75%^9^ and specific molecular genetic markers of ADHD have now been identified,^10^ a variety of environmental factors may also be associated with an increased risk of ADHD.^11,12^ Prenatal valproate exposure may be associated with ADHD, although previous studies showed no clear evidence of this.^4^ If so, this would be a modifiable environmental exposure and of major importance for women of childbearing potential using valproate.

In this large population-based prospective cohort study, we evaluated the association between maternal use of valproate and other AEDs during pregnancy and the risk of ADHD in the offspring, taking maternal history of epilepsy and psychiatric conditions into account.

## Methods

All data were analyzed at Statistics Denmark using encrypted identification numbers with no contact with the individuals. By Danish law, analyses of anonymous data do not require ethical review board approval. However, the study was approved by the Danish Data Protection Agency. Because the data were deidentified, informed consent was not required. The study follows the Strengthening the Reporting of Observational Studies in Epidemiology (STROBE) reporting guideline for cohort studies.

### Study Design and Study Population

We conducted a population-based cohort study and included in the study population all singleton children born alive in Denmark between January 1, 1997, and December 31, 2011. The cohort was followed up from birth until the day of the ADHD diagnosis (*International Classification of Diseases, Tenth Revision, Diagnostic Criteria for Research* [*ICD-10-DCR*] diagnosis and/or use of ADHD medication), death, emigration, or December 31, 2015, whichever came first. Data were analyzed in September 2018.

### Medication Exposure

In Denmark, every individual is assigned a unique personal identification number in the Danish Civil Registration System when he or she is born in or immigrates to Denmark.^13^ This ensured complete linkage of individual information in all national registries used in this study.

The Danish National Prescription Registry^14^ holds unique information on all redeemed prescriptions purchased by patients (medical treatment given only in hospitals is not included) since January 1, 1996. In Denmark, all antiepileptic prescriptions filled by patients are prescribed by hospital physicians or general practitioners. We defined the exposure window to the period from 30 days before the estimated day of conception to the day of birth, and included children with an estimated time of conception after February 1, 1996, ie, children born after January 1, 1997, and no later than December 31, 2011. Exposure to AEDs was defined as any redeemed prescriptions with the Anatomical Therapeutic Chemical code N03A (AEDs), including N03AG01 (valproate) or N05BA09 (clobazam), within the exposure window.

Monotherapy was defined as pregnancies in which the mothers had redeemed prescriptions for only 1 type of AED, and polytherapy was defined as pregnancies in which the mothers had redeemed prescriptions for more than 1 type of AED within the exposure window. For both monotherapy and polytherapy, the mothers may also have redeemed prescriptions for other types of medications than AEDs. Besides valproate, we also estimated the risk of ADHD among the offspring to mothers with other frequently used AEDs during pregnancy (carbamazepine, clonazepam, lamotrigine, and oxcarbazepine), because these AEDs were the most commonly used AEDs in Denmark during the study period.

The mean daily dose of AED was estimated from the total amount of AED redeemed from 30 days before pregnancy to birth, divided by the number of days in the same period.

### Information on ADHD and Covariates

The Danish Psychiatric Central Research Register^15^ was used to identify children who were diagnosed for the first time with ADHD, based on *ICD-10-DCR* codes F90 and F98.8. We also used the Danish National Prescription Registry^14^ to identify offspring who used medication that is used for ADHD treatment (N06BA01, amphetamine; N06BA02, dexamphetamine; N06BA04, methylphenidate; N06BA09, atomoxetine; N06BA11, dexmethylphenidate; and N06BA12, lisdexamfetamine). In Denmark, the vast majority (>70%) of children with ADHD are diagnosed in an outpatient clinic at a public hospital, free of charge.^16^ Most of the remaining children diagnosed with ADHD outside hospital departments, however, would be identified from filled prescriptions for ADHD medications.

Information on parity was obtained from the Danish Medical Birth Registry.^17^ We used the Danish National Patient Register^18^ to identify children diagnosed with congenital malformations (*ICD 10-DCR* codes Q0-Q99, except for undescended testicle [Q53] and congenital deformities of hip [Q65] because of the low validity of these diagnoses)^15^ and mothers diagnosed with epilepsy before the birth of the child (*International Classification of Diseases, Eighth Revision* [*ICD-8*] code 345 and *ICD-10-DCR* codes G40 and G41). We identified mothers diagnosed with psychiatric disorders before the birth of their child (*ICD-8* codes 290-315 and *ICD-10-DCR* codes F0.00-F99.9) from the Danish Psychiatric Central Research Register.

### Statistical Analysis

We used Cox regression to estimate the hazard ratio (HR), including 95% CI, for ADHD for children with prenatal valproate exposure, with the age of the child as the underlying time scale and separate baseline diagnostic rates (stratum) for each birth year group to adjust for the decreasing use of valproate in pregnancy and the increasing prevalence of ADHD. The proportional hazards assumption was evaluated for all variables by comparing estimated log[−log(survival)] curves over the different categories of variables investigated. Control for the lack of independence of children within the same family was obtained using a robust (Huber-White) variance estimator. The HRs were adjusted for risk factors for ADHD, including maternal age at conception (15-24, 25-29, 30-34, or ≥35 years), maternal psychiatric history (yes or no), maternal epilepsy (yes or no), maternal diabetes (yes or no), sex of the child, and parity (1, 2, or ≥3). We used competing risk regression to estimate the absolute risk (cumulative incidence) of ADHD in the first 15 years of life after prenatal valproate exposure in pregnancy.

To assess possible confounding by indication, we compared the risk of ADHD in offspring of women who used valproate during pregnancy with that of women who discontinued their use of valproate at least 30 days before the estimated date of conception (ie, previous users). This analysis addresses whether the illness that triggered valproate use rather than valproate exposure was associated with ADHD in the child. In sensitivity analyses, we also estimated the risk of ADHD after stratifying the risk according to dose of valproate, trimester of exposure (first vs later), monotherapy vs polytherapy, and maternal use of other AED, after adjusting for maternal smoking (yes or no) and after excluding children with congenital malformations. Further, we performed sensitivity analyses by excluding children with epilepsy and children whose mother had a diagnosis of ADHD and by increasing the exposure period before the estimated time of conception from 30 days to 90 days.

Finally, we estimated the HR of ADHD with follow up at 3 years of age. Data analyses were performed using Stata version 12 statistical software (StataCorp LP).

## Results

We identified 913 302 children born in Denmark from January 1, 1997, to December 31, 2011, who contributed more than 10.2 million person-years of observation (mean age at end of study, 10.1 years; median age, 9.4 years; interquartile range, 7.2-12.8 years; 468 708 [51.3%] male). In this cohort, 580 children had been exposed to valproate during pregnancy (monotherapy and polytherapy combined) and 912 722 had not. Table 1 shows the maternal and offspring characteristics of the study population according to exposure to valproate in pregnancy. Compared with mothers who did not use valproate during pregnancy, mothers who used valproate during pregnancy were younger, were more often diagnosed with epilepsy and psychiatric disorders, and were more often smokers during their pregnancy. Compared with unexposed children, children exposed to valproate were more often diagnosed with congenital malformations during their first year of life.

**Table 1.** Maternal and Offspring Characteristics of the Study Population of 913 302 Children Born in Denmark From January 1, 1997, to December 31, 2010, by Exposure to Valproate in Pregnancy

Characteristic	No. (%)	
	Prenatal Exposure to Valproate (n = 580)	No Prenatal Exposure to Valproate (n = 912 722)
**Maternal Characteristics**		
Age, y		
15-24	105 (18.1)	122 304 (13.4)
25-29	189 (32.6)	310 282 (34.0)
30-34	196 (33.8)	324 315 (35.5)
≥35	90 (15.5)	155 821 (17.1)
Epilepsy diagnosis		
No	64 (11.0)	905 618 (99.2)
Yes	516 (89.0)	7104 (0.8)
Psychiatric diagnosis		
No	506 (87.2)	841 309 (92.2)
Yes	74 (12.8)	71 413 (7.8)
Diabetes diagnosis		
No	564 (97.2)	889 727 (97.5)
Yes	16 (2.8)	22 995 (2.5)
Smoking		
No	343 (59.1)	674 865 (73.9)
Yes	118 (20.3)	143 633 (15.7)
Missing	119 (20.5)	94 224 (10.3)
Parity		
1	241 (47.3)	392 798 (43.0)
2	174 (34.1)	334 259 (36.6)
≥3	87 (17.1)	172 510 (18.9)
Missing	8 (1.6)	13 155 (1.4)
**Offspring Characteristics**		
Sex		
Male	302 (51.3)	468 406 (51.3)
Female	278 (48.7)	444 316 (48.7)
Birth year		
1997-1999	189 (32.6)	183 871 (20.1)
2000-2002	126 (21.7)	186 165 (20.4)
2003-2005	116 (20.0)	183 097 (20.1)
2006-2008	88 (15.2)	184 049 (20.2)
2009-2011	61 (10.5)	175 540 (19.2)
Malformations in first year of life		
No	512 (88.3)	873 710 (95.7)
Yes	68 (11.7)	39 012 (4.3)

A total of 29 445 persons were identified with ADHD (19 102 redeemed prescriptions of ADHD medications and 25 430 were diagnosed with ADHD in the Danish Psychiatric Central Research Register). Of the 912 722 children who were unexposed to valproate, 29 396 (3.2%) had ADHD. Of the 580 children who had been exposed to valproate, 49 (8.4%) had ADHD. The mean age at ADHD diagnosis in this study was 8.8 years (median age, 8.0 years; interquartile range, 6.0-11.0 years).

### Main Findings

Overall, the children who were prenatally exposed to valproate had a 48% increased risk of ADHD (adjusted HR [aHR], 1.48; 95% CI, 1.09-2.00) compared with the unexposed children (Table 2). The absolute 15-year risk of ADHD was 4.6% (95% CI, 4.5%-4.6%) in children unexposed to valproate and 11.0% (95% CI, 8.2%-14.2%) in children who were exposed to valproate in pregnancy.

**Table 2.** Hazard Ratio of ADHD in the Offspring of Women Who Used Valproate During Pregnancy

ADHD Diagnosis by Valproate Exposure	Live Births, No.	Person-Years at Risk	ADHD Diagnoses, No.	Incidence/1000 Person-Years (95% CI)	Hazard Ratio (95% CI)	
					Crude	Adjusted^a^
ADHD diagnosis based on medication and *ICD-10* diagnosis						
Exposed to valproate	580	7066	49	6.9 (5.2-9.2)	2.28 (1.72-3.02)	1.48 (1.09-2.00)
Not exposed to valproate	912 722	10 212 263	29 396	2.9 (2.8-2.9)	1 [Reference]	1 [Reference]
ADHD diagnosis based on medication alone						
Exposed to valproate	580	7166	30	4.2 (2.9-6.0)	2.08 (1.45-2.98)	1.29 (0.88-1.91)
Not exposed to valproate	912 722	10 260 068	19 072	1.9 (1.8-1.9)	1 [Reference]	1 [Reference]
ADHD diagnosis based on *ICD-10* diagnosis alone						
Exposed to valproate	580	7096	44	6.2 (4.6-8.3)	2.37 (1.76-3.18)	1.52 (1.10-2.10)
Not exposed to valproate	912 722	10 235 021	25 386	2.5 (2.4-2.5)	1 [Reference]	1 [Reference]

Abbreviations: ADHD, attention-deficit/hyperactivity disorder; *ICD-10*, *International Statistical Classification of Diseases and Related Health Problems, Tenth Revision*.

^a^ Adjusted for maternal age at conception, maternal psychiatric history, maternal epilepsy, maternal diabetes, sex of the child, year of birth, and parity.

When restricting the cohort to the 7620 children born to women with epilepsy, valproate use during pregnancy (n = 516) was associated with a 39% higher risk of ADHD in children (aHR, 1.39; 95% CI, 1.00-1.93) compared with the risk in children born to mothers with epilepsy who did not use valproate during pregnancy (n = 7104) (Table 3). The point estimate comparing children who were born to women without epilepsy and were exposed to valproate during pregnancy (n = 64) vs children born to mothers without epilepsy who did not use valproate during pregnancy (n = 905 618) showed no significant difference in risk of ADHD (aHR, 1.89; 95% CI, 0.76-4.68).

**Table 3.** Hazard Ratio of ADHD in the Offspring of Women Who Used Valproate During Pregnancy Stratified by Epilepsy Diagnosis in the Mother

Maternal Epilepsy Diagnosis	Valproate Exposure	Live Births, No.	Person-Years at Risk	ADHD Diagnoses, No.	Incidence/1000 Person-Years (95% CI)	Hazard Ratio (95% CI)	
						Crude	Adjusted^a^
Epilepsy	Exposed	516	6232	44	7.1 (5.3-9.5)	1.27 (0.93-1.73)	1.39 (1.00-1.93)
	Not exposed	7104	69 187	342	4.9 (4.4-5.5)	1 [Reference]	1 [Reference]
No epilepsy	Exposed	64	834	5	6.0 (2.5-14.4)	1.90 (0.78-4.60)	1.89 (0.76-4.68)
	Not exposed	905 618	10 143 077	29 054	2.9 (2.8-2.9)	1 [Reference]	1 [Reference]

Abbreviation: ADHD, attention-deficit/hyperactivity disorder.

^a^ Adjusted for maternal age at conception, maternal psychiatric history, maternal diabetes, sex of the child, year of birth, and parity.

In the offspring of women who used valproate in monotherapy during pregnancy (n = 431), the risk of ADHD was increased by 52% compared with the risk in the offspring of women who did not use AEDs during pregnancy (aHR, 1.52; 95% CI, 1.05-2.19) (Table 4 and Figure). Compared with the offspring unexposed to AEDs in pregnancy, the risk of ADHD associated with exposure to monotherapy with other AEDs was not statistically different (carbamazepine: aHR, 1.23; 95% CI, 0.84-1.82; clonazepam: aHR, 1.43; 95% CI, 0.95-2.16; oxcarbazepine: aHR, 1.10; 95% CI, 0.72-1.67; lamotrigine: aHR, 0.84; 95% CI, 0.59-1.19) (Table 4 and Figure).

**Table 4.** Hazard Ratio of ADHD in the Offspring of Women Who Used Antiepileptic Drugs in Monotherapy During Pregnancy Compared With the Risk in Offspring of Women Who Did Not Use Antiepileptic Drugs in Pregnancy

Monotherapy Exposure to Antiepileptic Drugs	Live Births, No.	Person-Years at Risk	ADHD Diagnoses, No.	Incidence/1000 Person-Years (95% CI)	Hazard Ratio (95% CI)	
					Crude	Adjusted^a^
Valproate	431	5307	38	7.2 (5.2-9.8)	2.37 (1.73-3.26)	1.52 (1.05-2.19)
Carbamazepine	423	5556	31	5.6 (3.9-7.9)	1.78 (1.25-2.54)	1.23 (0.84-1.82)
Clonazepam	314	3657	25	6.8 (4.6-10.1)	2.35 (1.59-3.47)	1.43 (0.95-2.16)
Oxcarbazepine	372	4577	25	5.5 (3.7-8.1)	1.81 (1.22-2.68)	1.10 (0.72-1.67)
Lamotrigine	1383	12 140	41	3.4 (2.5-4.6)	1.45 (1.07-1.98)	0.84 (0.59-1.19)
Not exposed	899 941	10 086 866	28 752	2.9 (2.8-2.9)	1 [Reference]	1 [Reference]

Abbreviation: ADHD, attention-deficit/hyperactivity disorder.

^a^ Adjusted for maternal age at conception, maternal psychiatric history, maternal diabetes, sex of the child, year of birth, and parity.

**Figure.**
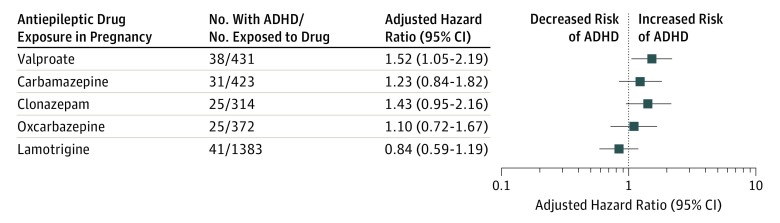
Adjusted Hazard Ratio of Attention-Deficit/Hyperactivity Disorder (ADHD) in the Offspring of Women Who Used Antiepileptic Drugs in Monotherapy During Pregnancy Adjusted for maternal age at conception, maternal psychiatric history, maternal epilepsy, maternal diabetes, sex of the child, year of birth, and parity.

When compared with prenatal exposure to lamotrigine (using this as the reference), the risk of ADHD associated with prenatal valproate exposure was more than 2-fold increased (aHR, 2.16; 95% CI, 1.34-3.48), carbamazepine increased the risk by 79% (aHR, 1.79; 95% CI, 1.06-3.04), and clonazepam increased the risk by 96% (aHR, 1.96; 95% CI, 1.09-3.50). Compared with the offspring exposed to lamotrigine in pregnancy, the risk of ADHD associated with prenatal exposure to oxcarbazepine was not significantly increased (aHR, 1.58; 95% CI, 0.91-2.58) (eTable 1 in the Supplement).

### Sensitivity Analyses

The risk of ADHD in the offspring of 580 women who used valproate in pregnancy was 66% higher than the risk of ADHD in the offspring of 719 women who used valproate prior to, but not during, pregnancy (aHR, 1.66; 95% CI, 1.05-2.62) (eTable 2 in the Supplement).

Among the 580 women who filled a prescription for valproate during pregnancy, 499 filled a prescription in the first trimester or later in pregnancy, whereas 81 filled a prescription for valproate after their first trimester only (ie, in their second or third trimester). Compared with the offspring of children who were unexposed to valproate, the risk of ADHD was 52% higher for exposure in the first trimester (aHR, 1.52; 95% CI, 1.10-2.10) (eTable 3 in the Supplement) and 22% higher for valproate exposure after the first trimester only (aHR, 1.22; 95% CI, 0.52-2.86) (eTable 3 in the Supplement).

The aHR of ADHD was 1.52 (95% CI, 1.05-2.19) in the offspring of 431 women who used valproate as monotherapy and 1.43 (95% CI, 0.75-2.70) in the offspring of 149 women who used valproate as polytherapy when compared with the offspring of 899 941 women who did not use an AED during pregnancy (eTable 4 in the Supplement).

Compared with the offspring of 899 941 unexposed women, the offspring of 204 women who were estimated to use valproate in high-dose monotherapy (>750 mg/d) had a higher risk of ADHD (aHR, 1.68; 95% CI, 1.04-2.71). However, the risk of ADHD did not significantly differ for the offspring of 227 women who were estimated to use valproate in low-dose monotherapy (≤750 mg) compared with the offspring of 899 941 unexposed women (aHR, 1.37; 95% CI, 0.82-2.27) (eTable 5 in the Supplement).

A large proportion of women had missing information on smoking during pregnancy (Table 1). When adjusting for maternal smoking, the risk of ADHD associated with valproate exposure in pregnancy was still significantly increased compared with unexposed mothers (aHR, 1.55; 95% CI, 1.10-2.19) (eTable 6 in the Supplement). Similarly, when excluding 39 080 children with congenital malformations, the risk of ADHD among 512 children prenatally exposed to valproate was also significantly increased (aHR, 1.47; 95% CI, 1.06-2.05) compared with 873 710 children not exposed to valproate (eTable 7 in the Supplement).

After excluding children with epilepsy, the children who were prenatally exposed to maternal valproate had an aHR of ADHD of 1.53 (95% CI, 1.05-2.23) compared with the unexposed children (eTable 8 in the Supplement). Sensitivity analyses were also performed for other AEDs used in monotherapy (eTable 9 in the Supplement).

After excluding mothers with ADHD, the children who were prenatally exposed to valproate had an aHR of ADHD of 1.56 (95% CI, 1.10-2.21) compared with the unexposed children (eTable 10 in the Supplement).

The valproate-exposed children who were born to mothers who used valproate 90 days before conception to birth had a 48% increased risk of ADHD (aHR, 1.48; 95% CI, 1.05-2.07) compared with the unexposed children (eTable 11 in the Supplement).

When follow-up was started at 3 years of age, children who were prenatally exposed to maternal valproate had an aHR of ADHD of 1.93 (95% CI, 1.28-2.91) compared with the unexposed children (eTable 12 in the Supplement). The aHRs of ADHD in children exposed to other AEDs used in monotherapy compared with unexposed children were similar to aHRs when follow-up was started at birth (eTable 12 in the Supplement). Age at the end of follow-up was similar for unexposed children (mean age, 10.9 years) and children exposed to the various AEDs (mean ages, 8.4-12.9 years) (eTable 12 in the Supplement).

## Discussion

This study identifies an association between prenatal exposure to valproate during pregnancy and the risk of ADHD in the offspring. The study therefore adds to the increasing number of studies suggesting that valproate in pregnancy is associated with a number of adverse neurodevelopmental outcomes, including poor cognitive function^3,19,20,21^ and autism,^22^ in addition to an increased risk of congenital malformations.^23^

The risk of ADHD was also increased in valproate-exposed offspring compared with the offspring of women who used valproate prior to but not during their pregnancy and compared with children who were prenatally exposed to lamotrigine.

The association between prenatal exposure to valproate and an increased risk of ADHD was quite robust and persisted after adjusting for maternal psychiatric disorders, maternal epilepsy, maternal diabetes, maternal age, sex, year of birth, and parity, and the association remained after excluding women who smoked during pregnancy.^24^ The association persisted when examining valproate dose and polytherapy, but the number of exposed children and the statistical precision were low; the results of this analysis as well as a number of sensitivity analyses discussed later should therefore be interpreted with caution. Valproate exposure was relatively rare in this cohort (approximately 63.5 children with valproate exposure per 100 000 children), and the association with offspring ADHD was modest. Thus, even in a cohort of this size, the additional number of children who would develop ADHD due to prenatal valproate exposure is low.

The Danish Psychiatric Central Research Register^15^ may not capture all mothers with psychiatric disorders (including ADHD) or misuse of alcohol, especially when intake is moderate. In addition, the Danish National Patient Register^18^ may not capture all women with epilepsy. Thus, we cannot exclude that the association between maternal valproate use in pregnancy and ADHD in the offspring may be, at least in part, due to unmeasured confounding.

The results run counter to a recent meta-analysis of 4 studies including a total of 750 individuals, which did not find an association between prenatal valproate exposure and offspring ADHD symptoms.^4^ Possible explanations for this discrepancy include differences in the cohorts analyzed as well as smaller sample sizes, higher attrition rates, and shorter follow-ups in the studies included in the meta-analysis than in the present study.^4^

Although not significant, analyses of other types of AEDs suggest that these AEDs may also carry a risk of ADHD in the offspring. For example, we found a possible risk increase associated with carbamazepine and clonazepam, which has not been previously described.^20,25^

The risk of ADHD was related to valproate exposure mainly in the first trimester, but the number of cases exposed only in later trimesters was low; our findings therefore do not exclude the possibility that valproate exposure across all stages of pregnancy may be associated with an increased risk of ADHD in the offspring. When examining doses of valproate, prenatal exposure to estimated higher doses tended to be associated with higher risks of ADHD than exposure to estimated lower doses of valproate, although the difference between high and low doses was not significant. Previous studies have indicated that the teratogenic and neurodevelopmental effects of valproate may be higher for high daily doses of valproate during pregnancy.^26^

We did not include socioeconomic status as a covariate in this study. Epilepsy in the mother may be associated with socioeconomic status and may thus be considered a mediator of the association between maternal epilepsy and offspring ADHD; thus, it should not be adjusted for in the estimate of the association between valproate exposure and risk of ADHD. In accordance with previous studies,^1,2,27^ we found that valproate exposure increased the risk of congenital malformations (Table 1). However, the risk of ADHD remained significant after adjusting for congenital malformations and after excluding all children diagnosed with congenital malformations from the analyses. This suggests that the increased ADHD risk was not confined to children with valproate-induced congenital malformations. The findings of this study add to the concerns related to use of valproate in pregnant women, and recently the European Medicines Agency and the US Department of Health and Human Services issued measures to avoid valproate exposure in pregnancy^28,29^ to protect the fetus. We did not have information on subtype of ADHD, which may offer clinically important insights into the symptoms in children with fetal valproate exposure.

A major strength of this study is its population-based nature and the completeness of follow-up without attrition. The study was able to combine data on ADHD diagnoses from hospital-based registers and data on the filling of prescriptions for drugs used in the treatment of ADHD. The analyses showed that most children who had been diagnosed with ADHD after prenatal valproate exposure were identified from both the hospital register and the prescription register, suggesting a high validity of both ways of identifying ADHD in children. Accordingly, several studies have validated the diagnosis of hyperkinetic disorders (*ICD-10-DCR* code F90) in the Danish Psychiatric Central Research Register and found that the risk of misclassification of patients is relatively low.^30^ Analyses of ADHD after prenatal valproate exposure based on hospital diagnoses and analyses based on filling of prescriptions for ADHD medication showed similar results (Table 2), although the estimate based on filled prescriptions did not reach statistical significance.

### Limitations

This study has important limitations to consider. First, pregnancy is a contraindication for valproate treatment. It is therefore likely that women who continue to be prescribed valproate during pregnancy differ in terms of disorder presentation and severity from women who do not. Several sensitivity analyses were conducted to try to address this limitation; however, due to the observational nature of the this study, we cannot rule out that the observed risk increase for ADHD is at least in part explained by the mother’s health condition that triggered the prescription of valproate during pregnancy. However, more than 50% of valproate-exposed children were born from 1997 through 2002 (Table 1), before it was widely recognized that valproate was associated with adverse birth outcomes—in particular, adverse neurodevelopmental outcomes—and at that time use of valproate was not contraindicated. The validity of the clinical ADHD diagnoses in the Danish Psychiatric Central Research Register has previously been shown to be high.^31^ However, clinical practice in Denmark may be more restrictive in diagnosing ADHD compared with other countries, such as the United States,^16^ and the hospital register and prescription register may include only the more severe ADHD cases. Thus, we may underestimate the full impact of valproate exposure in pregnancy and our findings might not be representative of all children exposed to valproate. Seizures may influence fetal development. However, it was not possible to identify pregnant women experiencing seizures from the registers.

Second, the data used in this study were based on register data, and we do not know whether the women used the medication and how much valproate was actually taken. However, for drugs used for chronic conditions, eg, AEDs, the agreement between self-reports and dispensing data are high.^32,33,34^ Furthermore, in the analyses of dose, we estimated the average daily dose on the basis of the total amount of redeemed prescriptions throughout pregnancy. It is unlikely that women would repeatedly purchase medication if it was not consumed, and it is therefore reasonable to assume that in the high-dose group, the proportion of women not actually consuming the medication would be low. The findings from the analyses considering dose are consistent with the main result.

Third, we did not control for other types of medications than AEDs that mothers may have redeemed during pregnancy, so we cannot rule out that part of the observed risk could be attributed to other medications beyond AEDs. In particular, information on the use of folic acid, which may be associated with neurodevelopment,^35,36^ was not included. However, including other drugs in the analyses may yield spurious associations due to the number, combinations, and variety of these drugs.

Fourth, to account for risk of epilepsy transmitted from the mother, we excluded children with epilepsy from the analyses, but this only had very limited impact on the risk estimate (eTable 8 and eTable 9 in the Supplement). Fifth, to account for risk of ADHD transmitted from the mother, we excluded women with ADHD from the analyses, although this only had very limited impact on the risk estimate. Sixth, we tried to account for misclassification of valproate exposure by increasing the exposure period before estimated conception to 90 days. However, this had almost no impact on the overall risk of ADHD in valproate-exposed children.

Seventh, symptoms of ADHD may not be recognizable before age 3 years, and we therefore included a sensitivity analysis in which follow-up started at age 3 years for each of the AEDs analyzed in monotherapy. However, this analysis also found an increased risk associated with valproate exposure in pregnancy and similar results for the other AEDs. Therefore, differences in follow-up do not account for the differences in risk of ADHD observed after exposure to the AEDs analyzed.

Eighth, despite the long follow-up period and nationwide nature of this study, several of the subgroup analyses for specific medications and patient groups had low power, as indicated by the wide confidence intervals. Therefore, findings must be interpreted with caution.

## Conclusions

Maternal use of valproate during pregnancy was associated with a small but significantly increased risk of ADHD in the offspring, even after adjusting for maternal psychiatric disease, maternal epilepsy, and other potential confounding covariates. These findings have important implications for the counseling of women of childbearing potential who are undergoing treatment with valproate, and they support warnings issued by authorities.^28,29^ As randomized clinical trials of valproate use during pregnancy are neither feasible nor ethical, our study provides clinical information on the risk of ADHD associated with valproate use during pregnancy. Replication of our findings in large-scale observational studies of adverse drug effects is warranted as such effects have not been evaluated adequately in controlled trials (ie, during pregnancy).

## Notes

Supplement. **eTable 1.** Hazard Ratio of Attention Deficit Hyperactivity Disorder (ADHD) in the Offspring of Women Who Used Antiepileptic Drugs in Monotherapy Compared With the Risk in the Offspring of Women Who Used Lamotrigine in Monotherapy During Pregnancy **eTable 2.** Hazard Ratio of Attention Deficit Hyperactivity Disorder (ADHD) in the Offspring of Women Who Used Valproate During Pregnancy Compared With the Risk in Offspring of Women Who Used Valproate Prior to Pregnancy **eTable 3.** Hazard Ratio of Attention Deficit Hyperactivity Disorder (ADHD) in the Offspring of Women Who Used Valproate During Pregnancy Stratified on Time of Exposure During Pregnancy **eTable 4.** Hazard Ratio of Attention Deficit Hyperactivity Disorder (ADHD) in the Offspring of Women Who Used Antiepileptic Drugs (AEDs) in Poly- and Monotherapy During Pregnancy Compared With the Offspring of Women Who Did Not Use Antiepileptic Drugs During Pregnancy **eTable 5.** Hazard Ratio of Attention Deficit Hyperactivity Disorder (ADHD) in the Offspring of Women Who Used Antiepileptic Drugs (AEDs) in Monotherapy at High and Low Dose During Pregnancy Compared With the Offspring of Women Who Did Not Use AEDs During Pregnancy **eTable 6.** Hazard Ratio of Attention Deficit Hyperactivity Disorder (ADHD) in the Offspring of Women Who Used Valproate During Pregnancy After Adjusting Also for Maternal Smoking **eTable 7.** Hazard Ratio of Attention Deficit Hyperactivity Disorder (ADHD) in Offspring of Women Who Used Valproate During Pregnancy After Excluding 39,080 Children With Congenital Malformations **eTable 8.** Hazard Ratio of Attention Deficit Hyperactivity Disorder (ADHD) in Offspring of Women Who Used Valproate During Pregnancy After Excluding 9,767 Children With Epilepsy **eTable 9.** Hazard Ratio of Attention Deficit Hyperactivity Disorder (ADHD) in the Offspring of Women Who Used Antiepileptic Drugs in Monotherapy During Pregnancy Compared With the Risk in Offspring of Women Who Did Not Use Antiepileptic Drugs in Pregnancy After Excluding 9,767 Children With Epilepsy **eTable 10.** Hazard Ratio of Attention Deficit Hyperactivity Disorder (ADHD) in the Offspring of Women Who Used Valproate During Pregnancy After Excluding 1,340 Women With ADHD From the Analyses **eTable 11.** Hazard Ratio of Attention Deficit Hyperactivity Disorder (ADHD) in the Offspring of Women Who Used Valproate During Pregnancy After Increasing the Exposure Period From 30 Days to 90 Before Conception **eTable 12.** Hazard Ratio of Attention Deficit Hyperactivity Disorder (ADHD) With Follow Up at 3 Years of Age in the Offspring of Women Who Used Antiepileptic Drugs in Monotherapy During Pregnancy Compared With the Risk in Offspring of Women Who Did Not Use Antiepileptic Drugs in Pregnancy
