# Error in Byline

**DOI:** 10.1001/jamanetworkopen.2019.1243

**Published:** 2019-02-22

In the Original Investigation titled “Association of Prenatal Exposure to Valproate and Other Antiepileptic Drugs With Risk for Attention-Deficit/Hyperactivity Disorder in Offspring,”^1^ published January 4, 2019, the second author’s name appeared incorrectly in the byline and should be Lars Pedersen, PhD. This article has been corrected.^1^
