# Supplementary material for: Association of Prenatal Exposure to Valproate and Other Antiepileptic Drugs With Risk for Attention-Deficit/Hyperactivity Disorder in Offspring
Source: JAMA Netw Open. 2019 Jan 4;2(1):e186606. doi: 10.1001/jamanetworkopen.2018.6606 (PMC6324310; doi:10.1001/jamanetworkopen.2018.6606)
Supplement: Supplement. — eTable 1. Hazard Ratio of Attention Deficit Hyperactivity Disorder (ADHD) in the Offspring of Women Who Used Antiepileptic Drugs in Monotherapy Compared With the Risk in the Offspring of Women Who Used Lamotrigine in Monotherapy During Pregnancy eTable 2. Hazard Ratio of Attention Deficit Hyperactivity Disorder (ADHD) in the Offspring of Women Who Used Valproate During Pregnancy Compared With the Risk in Offspring of Women Who Used Valproate Prior to Pregnancy eTable 3. Hazard Ratio of Attention Deficit Hyperactivity Disorder (ADHD) in the Offspring of Women Who Used Valproate During Pregnancy Stratified on Time of Exposure During Pregnancy eTable 4. Hazard Ratio of Attention Deficit Hyperactivity Disorder (ADHD) in the Offspring of Women Who Used Antiepileptic Drugs (AEDs) in Poly- and Monotherapy During Pregnancy Compared With the Offspring of Women Who Did Not Use Antiepileptic Drugs During Pregnancy eTable 5. Hazard Ratio of Attention Deficit Hyperactivity Disorder (ADHD) in the Offspring of Women Who Used Antiepileptic Drugs (AEDs) in Monotherapy at High and Low Dose During Pregnancy Compared With the Offspring of Women Who Did Not Use AEDs During Pregnancy eTable 6. Hazard Ratio of Attention Deficit Hyperactivity Disorder (ADHD) in the Offspring of Women Who Used Valproate During Pregnancy After Adjusting Also for Maternal Smoking eTable 7. Hazard Ratio of Attention Deficit Hyperactivity Disorder (ADHD) in Offspring of Women Who Used Valproate During Pregnancy After Excluding 39,080 Children With Congenital Malformations eTable 8. Hazard Ratio of Attention Deficit Hyperactivity Disorder (ADHD) in Offspring of Women Who Used Valproate During Pregnancy After Excluding 9,767 Children With Epilepsy eTable 9. Hazard Ratio of Attention Deficit Hyperactivity Disorder (ADHD) in the Offspring of Women Who Used Antiepileptic Drugs in Monotherapy During Pregnancy Compared With the Risk in Offspring of Women Who Did Not Use Antiepileptic Drugs in Pregnancy After Excluding [file jamanetwopen-2-e186606-s001.pdf]

Supplementary Online Content

Christensen J, Pedersen LH, Sun Y, Dreier JW, Brikell I, Dalsgaard S. Association of prenatal exposure to valproate and other antiepileptic drugs with risk for attention-deficit/hyperactivity disorder in offspring. *JAMA Netw Open*. 2019;2(1):e186606. doi:10.1001/jamanetworkopen.2018.6606

eTable 1. Hazard Ratio of Attention Deficit Hyperactivity Disorder (ADHD) in the Offspring of Women Who Used Antiepileptic Drugs in Monotherapy Compared With the Risk in the Offspring of Women Who Used Lamotrigine in Monotherapy During Pregnancy
eTable 2. Hazard Ratio of Attention Deficit Hyperactivity Disorder (ADHD) in the Offspring of Women Who Used Valproate During Pregnancy Compared With the Risk in Offspring of Women Who Used Valproate Prior to Pregnancy
eTable 3. Hazard Ratio of Attention Deficit Hyperactivity Disorder (ADHD) in the Offspring of Women Who Used Valproate During Pregnancy Stratified on Time of Exposure During Pregnancy
eTable 4. Hazard Ratio of Attention Deficit Hyperactivity Disorder (ADHD) in the Offspring of Women Who Used Antiepileptic Drugs (AEDs) in Poly- and Monotherapy During Pregnancy Compared With the Offspring of Women Who Did Not Use Antiepileptic Drugs During Pregnancy
eTable 5. Hazard Ratio of Attention Deficit Hyperactivity Disorder (ADHD) in the Offspring of Women Who Used Antiepileptic Drugs (AEDs) in Monotherapy at High and Low Dose During Pregnancy Compared With the Offspring of Women Who Did Not Use AEDs During Pregnancy
eTable 6. Hazard Ratio of Attention Deficit Hyperactivity Disorder (ADHD) in the Offspring of Women Who Used Valproate During Pregnancy After Adjusting Also for Maternal Smoking
eTable 7. Hazard Ratio of Attention Deficit Hyperactivity Disorder (ADHD) in Offspring of Women Who Used Valproate During Pregnancy After Excluding 39,080 Children With Congenital Malformations
eTable 8. Hazard Ratio of Attention Deficit Hyperactivity Disorder (ADHD) in Offspring of Women Who Used Valproate During Pregnancy After Excluding 9,767 Children With Epilepsy
eTable 9. Hazard Ratio of Attention Deficit Hyperactivity Disorder (ADHD) in the Offspring of Women Who Used Antiepileptic Drugs in Monotherapy During Pregnancy Compared With the Risk in Offspring of Women Who Did Not Use Antiepileptic Drugs in Pregnancy After Excluding 9,767 Children With Epilepsy
eTable 10. Hazard Ratio of Attention Deficit Hyperactivity Disorder (ADHD) in the Offspring of Women Who Used Valproate During Pregnancy After Excluding 1,340 Women With ADHD From the Analyses
eTable 11. Hazard Ratio of Attention Deficit Hyperactivity Disorder (ADHD) in the Offspring of Women Who Used Valproate During Pregnancy After Increasing the Exposure Period From 30 Days to 90 Before Conception
eTable 12. Hazard Ratio of Attention Deficit Hyperactivity Disorder (ADHD) With Follow Up at 3 Years of Age in the Offspring of Women Who Used Antiepileptic Drugs in

© 2019 Christensen J et al. *JAMA Network Open.*

Monotherapy During Pregnancy Compared With the Risk in Offspring of Women Who Did Not Use Antiepileptic Drugs in Pregnancy

This supplementary material has been provided by the authors to give readers additional information about their work.

© 2019 Christensen J et al. *JAMA Network Open.*

eTable 1. Hazard ratio of attention deficit hyperactivity disorder (ADHD) in the offspring of women who used antiepileptic drugs in monotherapy compared with the risk in the offspring of women who used lamotrigine in monotherapy during pregnancy

Monotherapy exposure to antiepileptic drugs	Live births (Number)	Person-years at risk	ADHD (Number)	Incidence (Cases per 1,000 person-years (95% CI)	Hazard ratio, crude (95% CI)		Hazard ratio, adjusted* (95% CI)	
Exposed to valproate	431	5,307	38	7.2 (5.2-9.8)	1.85	(1.21-2.84)	2.16	(1.34-3.48)
Exposed to carbamazepine	423	5,556	31	5.6 (3.9-7.9)	1.47	(0.92-2.33)	1.79	(1.06-3.04)
Exposed to clonazepam	314	3,657	25	6.8 (4.6-10.1)	1.76	(1.09-2.86)	1.96	(1.09-3.50)
Exposed to oxcarbazepine	372	4,577	25	5.5 (3.7-8.1)	1.40	(0.87-2.27)	1.58	(0.91-2.58)
Exposed to lamotrigine	1,383	12,140	41	3.4 (2.5-4.6)	1.0	(ref)	1.0	(ref)

*Adjusted for maternal age at conception, maternal psychiatric history, maternal epilepsy, maternal diabetes, sex of the child, year of birth and parity.

© 2019 Christensen J et al. *JAMA Network Open.*

eTable 2. Hazard ratio of attention deficit hyperactivity disorder (ADHD) in the offspring of women who used valproate during pregnancy compared with the risk in offspring of women who used valproate prior to pregnancy

Valproate exposure	Live births (Number)	Person-years at risk	ADHD (number)	Hazard ratio, crude (95% CI)		Hazard ratio, adjusted* (95% CI)	
During pregnancy	580	5,969	49	1.51	(1.00-2.28)	1.66	(1.05-2.62)
Prior to pregnancy	719	8,960	41		Ref	1.00	Ref

*Adjusted for maternal age at conception, maternal psychiatric history, maternal epilepsy, maternal diabetes, sex of the child, year of birth, and parity.

© 2019 Christensen J et al. *JAMA Network Open.*

eTable 3. Hazard ratio of attention deficit hyperactivity disorder (ADHD) in the offspring of women who used valproate during pregnancy stratified on time of exposure during pregnancy

Valproate exposure	Time of exposure	Live births (number)	Persons-years at risk	ADHD (number)	Hazard ratio, crude (95% CI)		Hazard ratio, adjusted* (95% CI)	
Valproate exposure	First trimester	499	6,120	43	2.31	(1.71-3.11)	1.52	(1.10-2.10)
	After first trimester only	81	946	6	2.12	(0.93-4.80)	1.22	(0.52-2.86)
No valproate exposure	Not exposed to valproate	912,722	10,212,263	29,396	1.00	(ref)	1.0	(ref)

*Adjusted for maternal age at conception, maternal psychiatric history, maternal epilepsy, maternal diabetes, sex of the child, year of birth, and parity.

© 2019 Christensen J et al. *JAMA Network Open.*

eTable 4. Hazard ratio of attention deficit hyperactivity disorder (ADHD) in the offspring of women who used antiepileptic drugs (AEDs) in poly- and monotherapy during pregnancy compared with the offspring of women who did not use antiepileptic drugs during pregnancy

AED	AED exposure	Live births (Number)	Person-years at risk	ADHD (Number)	Hazard ratio, crude (95% CI)		Hazard ratio, adjusted* (95% CI)	
Valproate	Monotherapy	431	5,307	38	2.37	(1.73-3.26)	1.52	(1.05-2.19)
	Polytherapy	149	1,1759	11	2.11	(1.17-3.83)	1.43	(0.75-2.70)
	No exposure to AEDs	899,941	10,086,866	28,752	1.0	(ref)	1.0	(ref)
Carbamazepine	Monotherapy	423	5,556	31	1.78	(1.25-2.54)	1.23	(0.84-1.82)
	Polytherapy	81	1,060	8	2.44	(1.21-4.92)	1.63	(0.78-3.41)
	No exposure to AEDs	899,941	10,086,866	28,752	1.0	(ref)	1.0	(ref)
Clonazepam	Monotherapy	314	3,657	25	2.35	(1.59-3.47)	1.43	(0.95-2.16)
	Polytherapy	130	1,464	11	2.64	(1.52-5.06)	1.70	(0.87-3.32)
	No exposure to AEDs	899,941	10,086,866	28,752	1.0	(ref)	1.0	(ref)
Lamotrigine	Monotherapy	1,383	12,140	41	1.45	(1.07-1.98)	0.84	(0.59-1.19)
	Polytherapy	363	3,711	24	2.43	(1.62-3.63)	1.39	(0.87-2.21)
	No exposure to AEDs	899,941	10,086,866	28,752	1.0	(ref)	1.0	(ref)
Oxcarbazepine	Monotherapy	372	4,577	25	1.81	(1.22-2.68)	1.10	(0.72-1.67)
	Polytherapy	120	1,401	9	2.17	(1.13-4.20)	1.37	(0.69-2.75)
	No exposure to AEDs	899,941	10,086,866	28,752	1.0	(ref)	1.0	(ref)

*Adjusted for maternal age at conception, maternal psychiatric history, maternal epilepsy, maternal diabetes, sex of the child, year of birth, and parity.

© 2019 Christensen J et al. *JAMA Network Open.*

eTable 5. Hazard ratio of attention deficit hyperactivity disorder (ADHD) in the offspring of women who used antiepileptic drugs (AEDs) in monotherapy at high and low dose during pregnancy compared with the offspring of women who did not use AEDs during pregnancy.

AED	AED exposure	Live births (Number)	Persons-years	ADHD(Number)	Hazard ratio, crude (95% CI)		Hazard ratio, adjusted* (95% CI)	
Valproate	High dose (>750 mg)	204	2,571	20	2.57	(1.66-3.96)	1.68	(1.04-2.71)
	Low dose (≤750 mg)	227	2,735	18	2.19	(1.37-3.49)	1.37	(0.82-2.27)
	No exposure	899,941	10,086,866	28,752	1.0	(ref)	1.0	(ref)
Carbamazepine	High dose (>500 mg)	245	3,284	15	1.45	(0.87-2.40)	0.99	(0.58-1.71)
	Low dose (≤500 mg)	178	2,272	16	2.29	(1.40-3.75)	1.55	(0.92-2.60)
	No exposure	899,941	10,086,866	28,752	1.0	(ref)	1.0	(ref)
Clonazepam	High dose (>4 mg)	26	365	<5	3.43	(1.33-8.85)	1.97	(0.76-5.14)
	Low dose (≤4 mg)	288	3,292	21	2.21	(1.44-3.39)	1.37	(0.87-2.14)
	No exposure	899,941	10,086,866	28,752	1.0	(ref)	1.0	(ref)
Lamotrigine	High dose (>150 mg)	895	7,918	21	1.13	(0.74-1.74)	0.68	(0.43-1.07)
	Low dose (≤150 mg)	488	4,222	20	2.08	(1.34-3.25)	1.08	(0.68-1.72)
	No exposure	899,941	10,086,866	28,752	1.0	(ref)	1.0	(ref)
Oxcarbazepine	High dose (>1,000 mg)	201	2,417	14	1.95	(1.15-3.31)	1.30	(0.75-2.28)
	Low dose (≤1,000 mg)	171	2,160	11	1.67	(0.93-3.00)	0.94	(0.52-1.70)
	No exposure	899,941	10,086,866	28,752	1.0	(ref)	1.0	(ref)

*Adjusted for maternal age at conception, maternal psychiatric history, maternal epilepsy, maternal diabetes, sex of the child, year of birth, and parity.

© 2019 Christensen J et al. *JAMA Network Open.*

eTable 6. Hazard ratio of attention deficit hyperactivity disorder (ADHD) in the offspring of women who used valproate during pregnancy after adjusting also for maternal smoking

Valproate exposure	Live births (Number)	Person-years at risk	ADHD (Number)	Incidence (per 1000 person-years) (95% CI)	Hazard ratio, crude (95% CI)		Hazard ratio, adjusted* (95% CI)	
Exposed to valproate	580	7,066	49	6.9 (5.2-9.2)	2.28	(1.72-3.02)	1.55	(1.10-2.19)
Not exposed to valproate	912,722	10,212,263	29,396	2.9 (2.8-2.9)	1.0	(ref)	1.0	(ref)

*Adjusted for maternal age at conception, maternal psychiatric history, maternal epilepsy, maternal diabetes, smoking, sex of the child, year of birth, and parity.

eTable 7. Hazard ratio of attention deficit hyperactivity disorder (ADHD) in offspring of women who used valproate during pregnancy after excluding 39,080 children with congenital malformations

(ADHD diagnosis in the offspring based on use of ADHD medicine and ICD 10 diagnosis)							
Valproate exposure	Live births (Number)	Person-years at risk	ADHD (Number)	Hazard ratio, crude (95% CI)		Hazard ratio, adjusted* (95% CI)	
Exposed to valproate	512	6,279	42	2.24	(1.65-3.03)	1.47	(1.06-2.05)
Not exposed to valproate	873,710	9,813,485	27,707	1.0	(ref)	1.0	(ref)

*Adjusted for maternal age at conception, maternal psychiatric history, maternal epilepsy, maternal diabetes, sex of the child, year of birth and parity.

© 2019 Christensen J et al. *JAMA Network Open.*

eTable 8. Hazard ratio of attention deficit hyperactivity disorder (ADHD) in offspring of women who used valproate during pregnancy after excluding 9,767 children with epilepsy

(ADHD diagnosis in the offspring based on use of ADHD medicine and ICD 10 diagnosis)							
Valproate exposure	Live births (Number)	Person-years at risk	ADHD (Number)	Hazard ratio, crude (95% CI)		Hazard ratio, adjusted* (95% CI)	
Exposed to valproate	519	6,200	40	2.20	(1.61-2.99)	1.53	(1.05-2.23)
Not exposed to valproate	903,016	10,095,537	28,297	1.0	(ref)	1.0	(ref)

*Adjusted for maternal age at conception, maternal psychiatric history, maternal epilepsy, maternal diabetes, sex of the child, year of birth and parity.

© 2019 Christensen J et al. *JAMA Network Open.*

eTable 9. Hazard ratio of attention deficit hyperactivity disorder (ADHD) in the offspring of women who used antiepileptic drugs in monotherapy during pregnancy compared with the risk in offspring of women who did not use antiepileptic drugs in pregnancy after excluding 9,767 children with epilepsy

Monotherapy exposure to antiepileptic drugs	Live births (Number)	Person-years at risk	ADHD (Number)	Incidence (Cases per 1,000 persons year) (95% CI)	Hazard ratio, crude (95% CI)		Hazard ratio, adjusted* (95% CI)	
Exposed to valproate	388	4,685	33	0.70 (0.50-0.99)	2.42	(1.72-3.39)	1.86	(1.21-2.85)
Exposed to carbamazepine	406	5,337	26	0.49 (0.33-0.72)	1.60	(1.09-2.36)	1.32	(0.83-2.10)
Exposed to clonazepam	307	3,573	23	0.64 (0.43-0.97)	2.27	(1.51-3.40)	1.36	(0.87-2.12)
Exposed to oxcarbazepine	355	4,347	20	0.46 (0.30-0.71)	1.57	(1.02-2.44)	1.08	(0.64-1.82)
Exposed to lamotrigine	1,353	11,814	39	0.33 (0.24-0.45)	1.47	(1.07-2.02)	0.92	(0.64-1.33)
Not exposed to antiepileptic drugs	890,532	9,973,474	27,702	0.28 (0.27-0.28)	1.0	(ref)	1.0	(ref)

*Adjusted for maternal age at conception, maternal psychiatric history, maternal diabetes, sex of the child, year of birth, and parity.

© 2019 Christensen J et al. *JAMA Network Open.*

eTable 10. Hazard ratio of attention deficit hyperactivity disorder (ADHD) in the offspring of women who used valproate during pregnancy after excluding 1,340 women with ADHD from the analyses

ADHD diagnosis based on medicine and ICD-10 diagnosis								
Valproate exposure	Live births (Number)	Person-years at risk	ADHD (Number)	Incidence (per 1,000 person-years) (95% CI)	Hazard ratio, crude (95% CI)		Hazard ratio, adjusted* (95% CI)	
Exposed to valproate	580	7,066	49	0.69 (0.52-0.92)	2.29	(1.73-3.03)	1.56	(1.10-2.21)
Not exposed to valproate	911,382	10,202,874	29,320	0.29 (0.28-0.29)	1.0	(ref)	1.0	(ref)

*Adjusted for maternal age at conception, maternal psychiatric history, maternal epilepsy, maternal diabetes, sex of the child, year of birth, and parity.

eTable 11. Hazard ratio of attention deficit hyperactivity disorder (ADHD) in the offspring of women who used valproate during pregnancy after increasing the exposure period from 30 days to 90 before conception

ADHD diagnosis based on medicine and ICD-10 diagnosis								
Valproate exposure	Live births (Number)	Person-years at risk	ADHD (Number)	Incidence (per 1,000 person-years) (95% CI)	Hazard ratio, crude (95% CI)		Hazard ratio, adjusted* (95% CI)	
Exposed to valproate	637	7,735	51	0.66 (0.50-0.87)	2.17	(1.65-2.86)	1.48	(1.05-2.07)
Not exposed to valproate	912,665	10,211,595	29,394	0.29 (0.28-0.29)	1.0	(ref)	1.0	(ref)

*Adjusted for maternal age at conception, maternal psychiatric history, maternal epilepsy, maternal diabetes, sex of the child, year of birth, and parity.

© 2019 Christensen J et al. *JAMA Network Open.*

eTable 12. Hazard ratio of attention deficit hyperactivity disorder (ADHD) with follow up at 3 years of age in the offspring of women who used antiepileptic drugs in monotherapy during pregnancy compared with the risk in offspring of women who did not use antiepileptic drugs in pregnancy

Monotherapy exposure to antiepileptic drugs	Live births (Number)	Person-years at risk	ADHD (Number)	Incidence (Cases per 1,000 persons year) (95% CI)	Hazard ratio, crude (95% CI)		Hazard ratio, adjusted* (95% CI)		Mean age at end of follow up (years)
Exposed to valproate	419	3.615	38	1.05 (0.76-1.44)	2.44	(1.78-3.36)	1.93	(1.28-2.91)	12.1
Exposed to carbamazepine	413	3.889	28	0.72 (0.50-1.04)	1.67	(1.15-2.41)	1.34	(0.86-2.10)	12.9
Exposed to clonazepam	306	2.419	23	0.95 (0.63-1.43)	2.22	(1.48-3.34)	1.27	(0.80-2.00)	11.4
Exposed to oxcarbazepine	367	3.102	25	0.81 (0.54-1.19)	1.86	(1.26-2.76)	1.17	(0.72-1.91)	11.9
Exposed to lamotrigine	1.355	6.681	37	0.55 (0.40-0.76)	1.38	(1.00-1.91)	0.84	(0.58-1.22)	8.4
Not exposed to antiepileptic drugs	883.311	6,527,235	27.832	0.43(0.42-0.43)	1.0	(ref)	1.0	(ref)	10.9

*Adjusted for maternal age at conception, maternal psychiatric history, maternal diabetes, sex of the child, year of birth, and parity.

© 2019 Christensen J et al. *JAMA Network Open.*
